# Intralobar sequestration of lung

**DOI:** 10.4103/0970-2113.56357

**Published:** 2009

**Authors:** R. Prasad, Rajiv Garg, Sanjay Kumar Verma

**Affiliations:** *Department of Pulmonary Medicine, Chhatrapati Sahuji Maharaj Medical University (erstwhile, King George's Medical University), Uttar Pradesh, Lucknow, India*

**Keywords:** Intralobar, lung, sequestration

## Abstract

Intralobar pulmonary sequestration is characterized by the presence of nonfunctional parenchymal lung tissue, receiving systemic arterial blood supply. It lacks normal communication with tracheobronchial tree. Failure to diagnose and treat this condition can lead to recurrent pneumonia and fatal hemoptysis. The aim of this case report is to increase awareness about the condition and to review criteria of its definitive diagnosis and subsequent treatment.

## INTRODUCTION

Bronchopulmonary sequestration is a benign, rare lung abnormality. It is characterized by the presence of a mass which is separated from normal bronchopulmonary tree. Anatomically it is classified into intralobar and extralobar sequestration; intralobar sequestration is more common and accounts for 0.15% to 1.7% of all congenital lung abnormalities. This case is reported because of its rare occurrence.

## CASE REPORT

**Figure 1:**
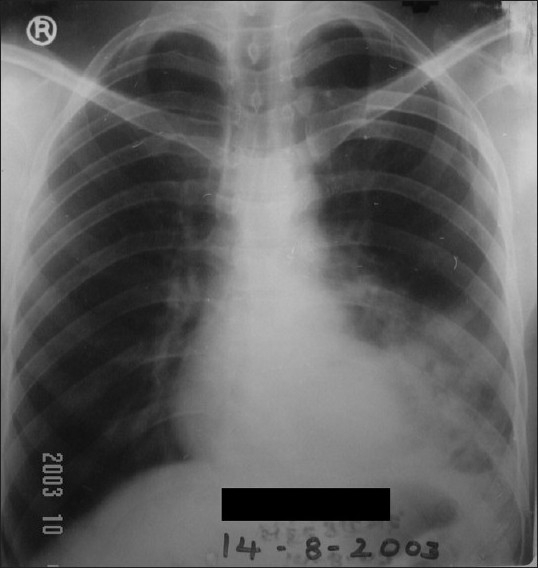
Chest X-ray revealing double-contour of the left cardiac border and inhomogeneous infiltrates on the left lower zone

**Figure 2:**
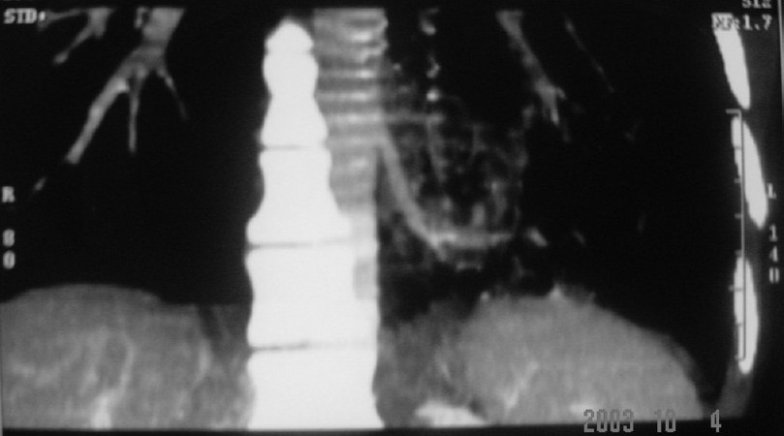
Aortography revealing anterobasal and posteromediobasal segments of left lower lobe, perfused via a single feeding vessel originating from descending thoracic aorta

A 35-year-old man, a nonsmoker, was admitted to our department with complaints of recurrent hemoptysis and cough with expectoration for four years. In the past, he had received antitubercular treatments without any clinico-radiological improvement. His resting pulse rate was 102/min, blood pressure - 112/74 mmHg, and respiratory rate - 26/min. His general examination revealed no significant abnormality. His respiratory system examination revealed coarse crepitations over the basal part of the left hemithorax. His chest X-ray revealed double-contour of the left cardiac border and inhomogeneous infiltrates on the left lower zone [Figure 1]. His blood examination showed normal hemogram. The result of his tuberculin skin test was negative. His sputum was negative for acid-fast bacilli (AFB) on three consecutive days. During hospital stay, the patient improved clinically after appropriate course of antibiotics, but opacity in the left lower zone persisted. He was further investigated, and his CT thorax was done. It revealed a cavity with fluid level localized to left lower anterobasal and posteromediobasal segments. Hence a possibility of lung sequestration, along with other possibilities, was raised. His aortography was done. It revealed that the anterobasal and posteromediobasal segments of left lower lobe were perfused via a single feeding vessel originating from descending thoracic aorta [Figure 2]. His selective angiography revealed an arterial supply from descending thoracic aorta, and venous drainage occurred via pulmonary veins. Hence a diagnosis of intralobar pulmonary sequestration was made. He was subjected to surgery with a right-sided double-lumen endotracheal tube for selective ventilation to achieve left-sided thoracotomy. The feeding vessel originating from descending thoracic aorta was ligated first and then the sequestrated part of left lower lobe segment was resected. Postoperative period was uneventful. Histopathology confirmed the diagnosis of intralobar sequestration (ILS).

## DISCUSSION

Pulmonary sequestration was first described by Rektorzik in 1861, as a malformation comprised of dysplastic lung tissue with no normal communication with the tracheobronchial tree and with an anomalous systemic arterial supply.[1] There are two types of pulmonary sequestrations: Intralobar sequestration (ILS), which is surrounded by normal lung tissue;[2] and extralobar sequestration (ELS), which has its own pleural investment. Intrapulmonary sequestration is four times more common than the extralobar type. The origin of intralobar sequestration was described in the past as being congenital and was explained by the accessory lung bud theory.[4–8] But the current widely held theory is that ILS is acquired after one or more episodes of necrotizing pneumonia resulting in obliterative bronchitis and obstruction of a lower lobe bronchus.

Most of the ILSs are located in the medial and posterior basal segments of the left lung. Overall, 98% occur in the lower lobes.[9] Bilateral involvement is uncommon. Associated congenital anomalies in ELS include diaphragmatic hernia, congenital cystic adenoid malformation, bronchogenic cysts, cardiovascular malformation, and pectus excavatum.[10–11] These are rare in intrapulmonary sequestrations. In ILS, the systemic arterial supply is via the descending thoracic aorta (72%), as seen in the present case; abdominal aorta, celiac axis, or splenic artery (21%); intercostal artery (3.7%); and rarely via the subclavian, internal thoracic, and pericardiophrenic arteries. Most venous drainage (95%) is via the pulmonary veins. The clinical hallmarks of ILS are recurrent cough with expectoration and hemoptysis.[12]

Chest radiographs can provide a reasonable diagnostic clue to pulmonary sequestration. A mass in the posterobasal segment of the lung in young patients with recurrent localized pulmonary infections is suggestive of intralobar sequestration (as in our case). In the past, aortography was frequently used for diagnosis. However, more recently, CT scan with contrast or MR angiography has been found to be easier and more useful.[13–17] The gold standard for identifying pulmonary sequestration is angiography as it confirms the anatomy, identifies the systemic supply, and shows the venous drainage.[3]

Management of an asymptomatic pulmonary sequestration with no connection to the surrounding lung is controversial. At present open surgery via posterolateral thoracotomy (PLT) remains the best established approach for definitive resection of bronchopulmonary sequestration, as has been done in the present case. The wide access by this approach facilitates the safe isolation and division of any abnormal systemic feeding arteries. However, video-assisted thoracic surgery (VATS) is now increasingly recognized as an equally effective, minimally invasive approach for bronchopulmonary sequestration.

In the current scenario of our country, where a lot of misuse of antibiotics and other drugs is quite prevalent, correct and prompt suspicion of nonresolving radiological lesions is needed to provide correct and early diagnosis of bronchopulmonary sequestration.
